# Phenotypic Changes on Mycobacterium Tuberculosis-Specific CD4 T Cells as Surrogate Markers for Tuberculosis Treatment Efficacy

**DOI:** 10.3389/fimmu.2018.02247

**Published:** 2018-09-28

**Authors:** Mohamed I. M. Ahmed, Nyanda E. Ntinginya, Gibson Kibiki, Bariki A Mtafya, Hadija Semvua, Stellah Mpagama, Charles Mtabho, Elmar Saathoff, Kathrin Held, Rebecca Loose, Inge Kroidl, Mkunde Chachage, Ulrich von Both, Antelmo Haule, Anna-Maria Mekota, Martin J. Boeree, Stephen H. Gillespie, Michael Hoelscher, Norbert Heinrich, Christof Geldmacher

**Affiliations:** ^1^Division of Infectious Diseases and Tropical Medicine, Klinikum of the University of Munich (LMU), Munich, Germany; ^2^German Center for Infection Research (DZIF), Partner Site Munich, Munich, Germany; ^3^CIH LMU Center for International Health, Klinikum of the University of Munich, Munich, Germany; ^4^National Institute for Medical Research—Mbeya Medical Research Center, Mbeya, Tanzania; ^5^Kilimanjaro Christian Medical Centre, Kilimanjaro Clinical Research Institute, Moshi, Tanzania; ^6^Dr. von Hauner Children's Hospital, Division of Paediatric Infectious Diseases, Klinikum of the University of Munich (LMU), Munich, Germany; ^7^Department of Lung Diseases, Radboud University Medical Centre Nijmegen, Nijmegen, Netherlands; ^8^Infection and Global Health Research Division, University of St Andrews, St Andrews, United Kingdom

**Keywords:** TAM-TB assay, tuberculosis, treatment monitoring, Mycobacterium tuberculosis-specific T cells, serial sputum culture, biomarker

## Abstract

**Background:** The analysis of phenotypic characteristics on *Mycobacterium tuberculosis* (MTB)-specific T cells is a promising approach for the diagnosis of active tuberculosis (aTB) and for monitoring treatment success. We therefore studied phenotypic changes on MTB-specific CD4 T cells upon anti-tuberculosis treatment initiation in relation to the treatment response as determined by sputum culture.

**Methods:** Peripheral blood mononuclear cells from subjects with latent MTB infection (*n* = 16) and aTB (*n* = 39) at baseline, weeks 9, 12, and 26 (end of treatment) were analyzed after intracellular interferon gamma staining and overnight stimulation with tuberculin. Liquid sputum cultures were performed weekly until week 12 and during 4 visits until week 26.

**Results:** T cell activation marker expression on MTB-specific CD4 T cells differed significantly between subjects with aTB and latent MTB infection with no overlap for the frequencies of CD38^pos^ and Ki67^pos^ cells (both *p* < 0.0001). At 9 weeks after anti-TB treatment initiation the frequencies of activation marker (CD38, HLA-DR, Ki67) positive MTB-specific, but not total CD4 T cells, were significantly reduced (*p* < 0.0001). Treatment induced phenotypic changes from baseline until week 9 and until week 12 differed substantially between individual aTB patients and correlated with an individual's time to stable sputum culture conversion for expression of CD38 and HLA-DR (both *p* < 0.05). In contrast, the frequencies of maturation marker CD27 positive MTB-specific CD4 T cells remained largely unchanged until week 26 and significantly differed between subjects with treated TB disease and latent MTB infection (*p* = 0.0003).

**Discussion:** Phenotypic changes of MTB-specific T cells are potential surrogate markers for tuberculosis treatment efficacy and can help to discriminate between aTB (profile: CD38^pos^, CD27^low^), treated TB (CD38^neg^, CD27^low^), and latent MTB infection (CD38^neg^, CD27^high^).

## Introduction

Novel diagnostic tools for improved detection of active tuberculosis (aTB) and for monitoring TB treatment are urgently required to succeed in the WHO END TB strategy, which—in the abscence of an efficacious MTB vaccine - sets the ambitious target of a world free of tuberculosis by 2030 [1]. Recent studies have highlighted the diagnostic potential of a flow cytometry based approach to detect and differentiate aTB disease from latent *Mycobacterium tuberculosis* (MTB) infection (LTBI) via phenotypic and/or functional characterization of MTB-specific T cells in adults and children (2–12). These “T cell activation and maturation marker assays” (TAM-TB assay) are sputum-independent, use easy-to-collect peripheral blood and—in contrast to the traditional immunodiagnostic Tuberculin skin test or Interferon gamma release assays (13)—allow highly specific detection of aTB (3, 5). TAM-TB assay results have been correlated with MTB loads in sputum (4, 9), with disease severity and with lung tissue destruction (4). Our previous study showed highly specific detection of childhood aTB in an endemic setting (3), potentially superior to sputum culture. Furthermore, TB treatment initiation decreases activation marker expression on MTB-specific CD4 T cells, probably reflecting the decrease of mycobacterial burden *in vivo* (5); which would make this a promising candidate marker for assessing TB treatment success.

While liquid culture and PCR are held to be the most sensitive tools to detect MTB, their widespread implementation for diagnosis and treatment monitoring is hampered by practical and methodological problems. Firstly, since these methods function by direct detection of the pathogen, they often remain false negative in paucibacillery aTB patients (14–16), and those where aTB lesions do not have access to the airways. As a consequence, TB treatment is often started on a presumptive diagnosis (17). Secondly, culture and PCR have shortcomings for monitoring of the TB treatment response. MTB culture methods have low sensitivity for unfavorable outcome and low positive predictive value estimates (18). The GeneXpert PCR shows a lag of positivity most likely due to detection of dead bacilli (19). The current treatment duration is that of a “one-size-fits-all” 6-months drug regimen without modifications based on treatment response monitoring. Past trials conducted by the MRC East Africa, and the more recent fluoroquinolone phase 3 studies, have demonstrated that more than 80% of TB patients will achieve cure after only 4 months of treatment (20–23). However to introduce a 4-months treatment as a blanket approach, it will be essential to discriminate between aTB patients who achieve cure already after 4 months and those in need of longer treatment. Sequential sputum bacterial load measurements by culture have been tested in this regard, but have insufficient sensitivity for detection of unfavorable treatment outcome on an individual basis (19, 24). Together, these shortcomings in mycobacteriological detection methods can impede accurate diagnosis of aTB, meaningful TB treatment monitoring and safe individualized treatment (21–23). The novel TAM-TB assay approach could potentially improve TB diagnosis and treatment monitoring; and hence help to overcome some of the challenges affecting diagnosis solely based on the direct detection of MTB bacilli in sputum. A prerequisite, herefore is a more detailed understanding of the relationship between TAM-TB assay results, the MTB infection status, and mycobacterial treatment response.

Here, we have therefore studied activation (CD38, HLA-DR, and Ki67) and maturation (CD27) marker profiles on IFNγ+ MTB-specific CD4 T cells in subjects with LTBI, and in aTB patients (25) before and after TB treatment initiation in comparison to the mycobacteriological treatment response. The patients were tightly monitored using MGIT culture on a weekly basis until week 12 and on 4 additional time points until the end of treatment at week 26 and showed no relapse during a 6 months follow-up after the end of treatment.

## Materials and methods

### Study populations, study samples, and ethics statements

**Figure 1:**
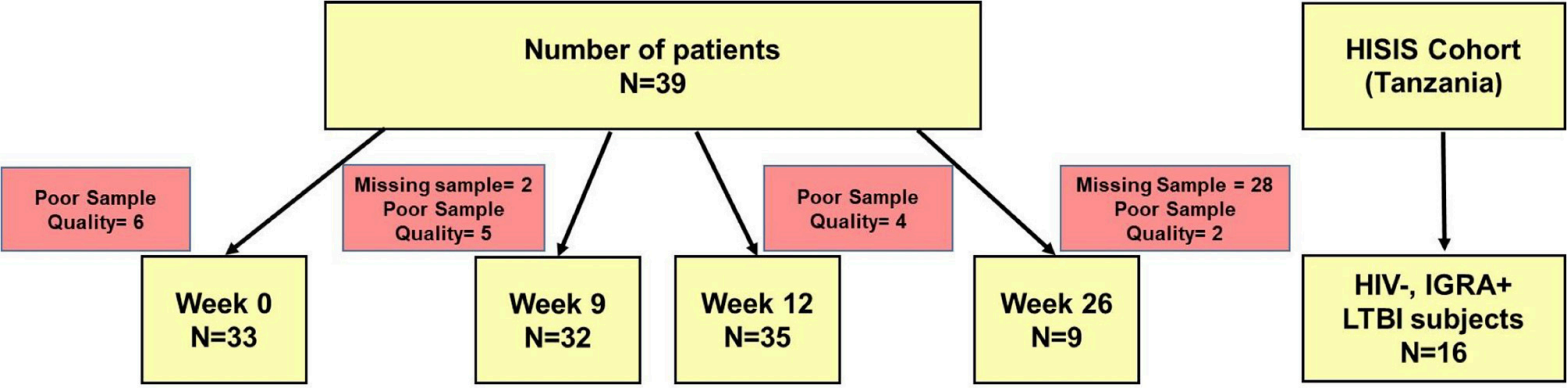
Diagram of study subjects, time points and TAM-TB analyses. Peripheral blood mononuclear cell samples from aTB patients (*n* = 39) were collected at baseline, at week 9, 12, and 26 after TB treatment initiation and subjected to TAM-TB assay analyses. The numbers of missing samples and those excluded due to none responsiveness to the positive control antigen SEB (“poor quality”) are indicated for each time point. PBMC samples (*n* = 16) from HIV- IGRA+ subjects were collected during the HISIS cohort study.

HIV^neg^ adult patients with culture confirmed aTB were enrolled at two clinical sites in Tanzania (NIMR-MMRC, Mbeya, and KCRI, Moshi) within the Panacea-TB MAMS treatment study (Table 1, Figure 1; clinicaltrials.gov identifier NCT01785186) (26). Four experimental treatments or the standard TB treatment were given for 12 weeks, followed by 8 weeks of standard treatment (rifampicin, isoniazid, pyrazinamide, and ethambutol) and by standard dose rifampicin and isoniazid to complete 26 weeks of treatment. All study participants received further follow-up for 6 months after end of treatment by telephonic interviews and site visits. Of the patients included in this sub-study, 25 were in either one of the experimental treatment arms, whereas 14 subjects received the standard treatment. None of the patients relapsed during this 6-months follow-up. The protocol was approved by independent ethics committees of the sponsor, the trial sites, and the regulatory authorities of Tanzania and South Africa. Additional samples from HIV^neg^ IGRA+ healthy female bar workers from the HISIS study (27) were included as LTBI controls (*n* = 16). The HISIS study was conducted at the NIMR-MMRC in compliance with national guidelines and institutional policies, and informed consent was obtained in accordance with the Declaration of Helsinki. The study was approved by the local ethic board at Mbeya (FWA no. 00002469) and the National Ethic Board at the National Institute for Medical Research (FWA no. 00002632).

**Table 1:** Demography of study subjects.

	**PanACEA**	**HISIS**
Gender (Male/Female)	30/9	0/16
Median Age (Range)	34.4 (19.2–65.4)	32 (17.9–38)
Median BMI (Range)	19.2 (15.2–41.6)	not determined
HIV status (+/–)	0/39	0/16
AFB sputum smear positivity grade (1/2/3)	3/10/26	Not applicable
Median Days to Positivity Baseline (Range)	3.5 (1–7.5)	Not applicable

### TAM-TB assay

Cryopreserved Peripheral Blood Mononuclear Cell (PBMC) samples from aTB patients (*n* = 39, Figure 1) from baseline, 9, 12, and 26 weeks were analyzed using the TAM-TB assay approach. PBMC samples from subjects with LTBI (*n* = 16) were included as additional controls. PBMCs were stimulated overnight at 37°C and 5% CO_2_ with Purified Protein Derivative (PPD, 10 μg/ml, Serum Staten Institute), SEB (0.6 μg/ml, Sigma-Aldrich) as a positive control, or no added peptide as negative control in the presence of Brefeldin A (BFA, final concentration 5 μg/ml, Sigma) and the costimulatory antibodies anti-CD49d (L25, BD) and anti-CD28 (L293, BD). Cells were stained with anti-CD38 BV785 (clone HIT2, Biolegend), anti-CD4 APC (clone 13B8.2, Beckmann Coulter), anti-CD27 ECD (clone 1A4CD27, Beckmann Coulter), and anti-HLA-DR APC-H7 (clone G46-6, Beckmann Dickinson), followed by fixation and permeabilization using FoxP3 Perm/Fix buffer and diluent (eBioscience), and then stained intracellularly using anti-IFNγ FITC (clone B27, BD Pharmingen), anti-Ki67 BV421 (clone B56, BD Pharmingen), and anti-CD3 APC-A700 (clone UCHT1, Beckmann Coulter). Cells were acquired on a CytoFlex Flow cytometer (Beckman Coulter). Data analysis was performed using FlowJo_V10. MTB-specific CD4 T cell responses were defined by a frequency of ≥0.03% of IFNγ+ CD4 T cells after PPD stimulation and by ≥2-fold increase over the negative control. Furthermore, a cell count of greater than 25 IFNγ+ CD4 T cell events had to be recorded. Samples with no response to the positive control antigen Staphylococcal enterotoxin B (SEB) were excluded from the analyses (*n* = 17). Pestle and Spice software (28)were used to analyze combinatorial expresssion of the four phenotypic markers on IFNγ+ MTB-specific CD4 T cells.

### Bacteriological assessments

Patients submitted sputum during weekly visits until week 12, and at weeks 14, 17, 22, and 26. Sputum was decontaminated with NaCl-OH, and cultured in liquid media; the mycobacterial growth indicator tube (Bactec MGIT960), and on Löwenstein-Jensen (LJ) solid medium (22). The primary study endpoint was time from treatment initiation to the first of two consecutive negative weekly sputum cultures without an intervening positive or contaminated culture in liquid media.

### Statistical analysis

The Statistical analysis was performed using GraphPad prism software version 6. The tests used are indicated in the Figure Legends.

## Results

### T cell activation marker expression profiles on MTB-specific CD4T cells differentiate between aTB and LTBI

**Figure 2:**
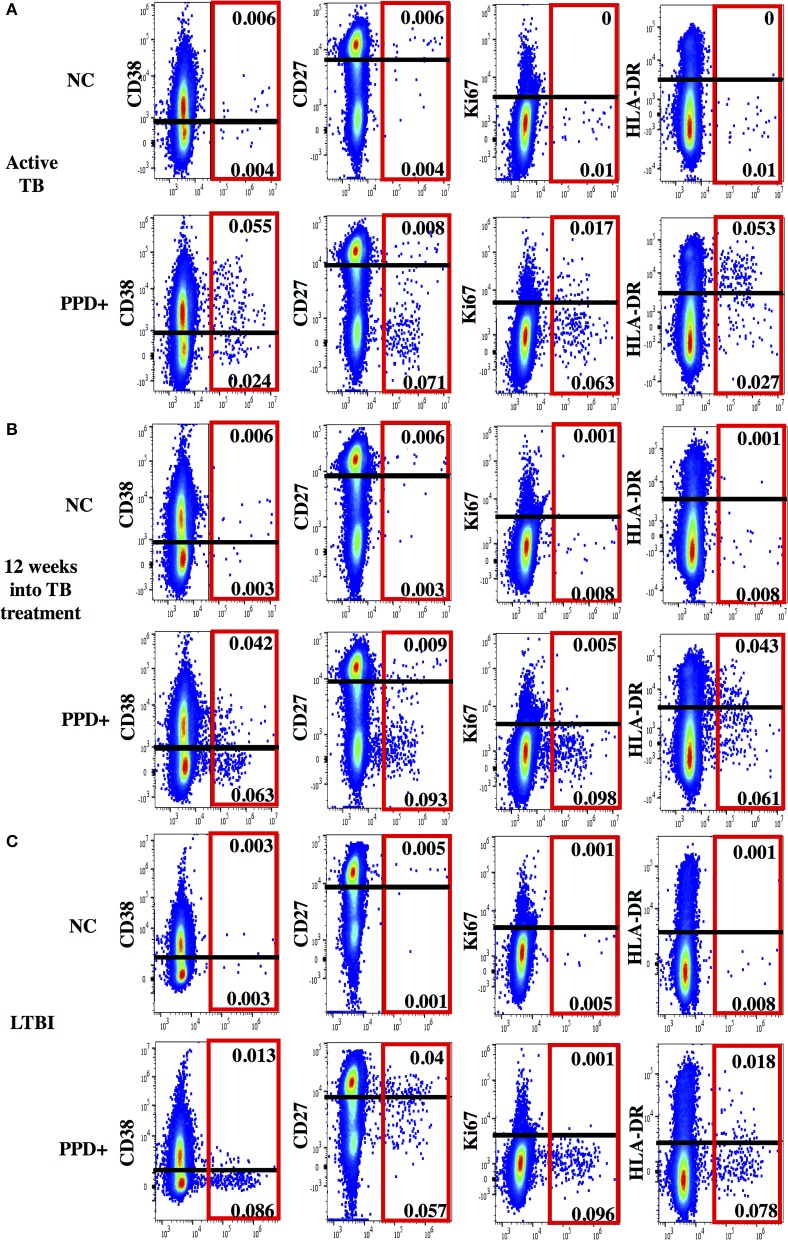
Representative dot plots for phenotypic characterization of MTB-specific CD4 T cells. Shown are dot plots for active TB **(A)**, 12 weeks into TB treatment **(B)** and Latent TB Infection **(C)**. Dot plots are gated on CD4 T cells showing IFNγ (x-axis) and activation (CD38, HLA-DR, and Ki67) and maturation (CD27) marker staining (y-axis) without stimulation (upper panel) and after PPD stimulation (lower panel). IFNγ+ MTB-specific CD4 T cells are indicated (red box). The cut-off for the expression of each phenotypic marker is indicated as a black line.

**Figure 3:**
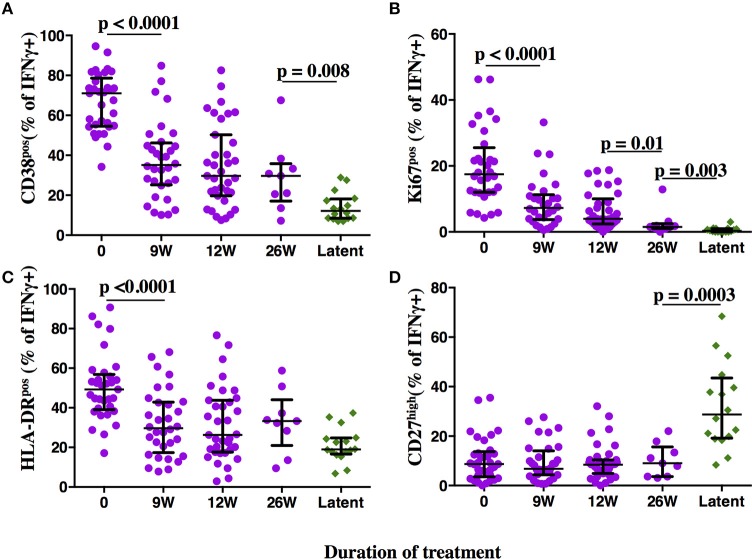
Phenotypic profiles of MTB-specific CD4 T cells in subjects with aTB, after TB treatment initiation and during LTBI. The frequency of MTB-specific CD4 T cells expressing the activation markers CD38 **(A)**, Ki67 **(B)**, HLA-DR **(C)**, and the maturation marker CD27 **(D)** is shown on the y-axis for pulmonary TB patients (purple circles) at baseline, 9, 12, and 26 weeks (x-axis) after TB treatment initiation. Subjects with latent MTB infection were included as controls (green diamonds). MTB-specific CD4 T cells were characterized after PPD stimulation. Statistical analyses were performed using the Mann-Whitney test. Median values, interquartile range and *p*-values below 0.05 are indicated.

At time of diagnosis, all aTB patients had detectable MTB-specific CD4 T cell responses upon PPD re-stimulation (median: 0.22%, range 0.03–4.3%). None-stimulated controls showed no or very little background in IFNγ^+^ T cells (median: 0.008%, range 0.0–0.066%). SEB stimulated controls showed high frequencies of IFNg+ CD4 T cells (median: 1.92%, range: 0.6–9.3%). High frequencies of MTB-specific CD4 T cells expressed the activation markers CD38 (median: 71%, Figure 3, representative dot plots in Figure 2), HLA-DR (median: 49.3%) and Ki67 (median: 17.5%) with a predominance of CD27^low^ cells (median: 91.3%), upon PPD stimulation. TAM expression on MTB-specific CD4 T cells differed significantly and showed no overlap for the frequencies of CD38^pos^ and Ki67^pos^ cells between aTB and LTBI (both *p* < 0.0001). Frequencies of CD27^high^ and HLA-DR^pos^ cells also differed (both *p* < 0.0001), but showed greater overlaps between aTB and LTBI. Receiver Operating Characteristic (ROC) curve analysis between aTB and LTBI (Supplementary Figure 1) confirmed that the frequency of CD38^pos^ and Ki67^pos^ MTB-specific CD4 T cells differentiated best between aTB and LTBI [Area under the Curve (AUC) = 1] with a defined cut-off of 31.55% for CD38 and 3.7% for Ki67 (Supplementary Figure 1).

### T cell activation marker expression profiles on MTB-specific CD4T cells change rapidly upon TB treatment initiation

**Figure 4:**
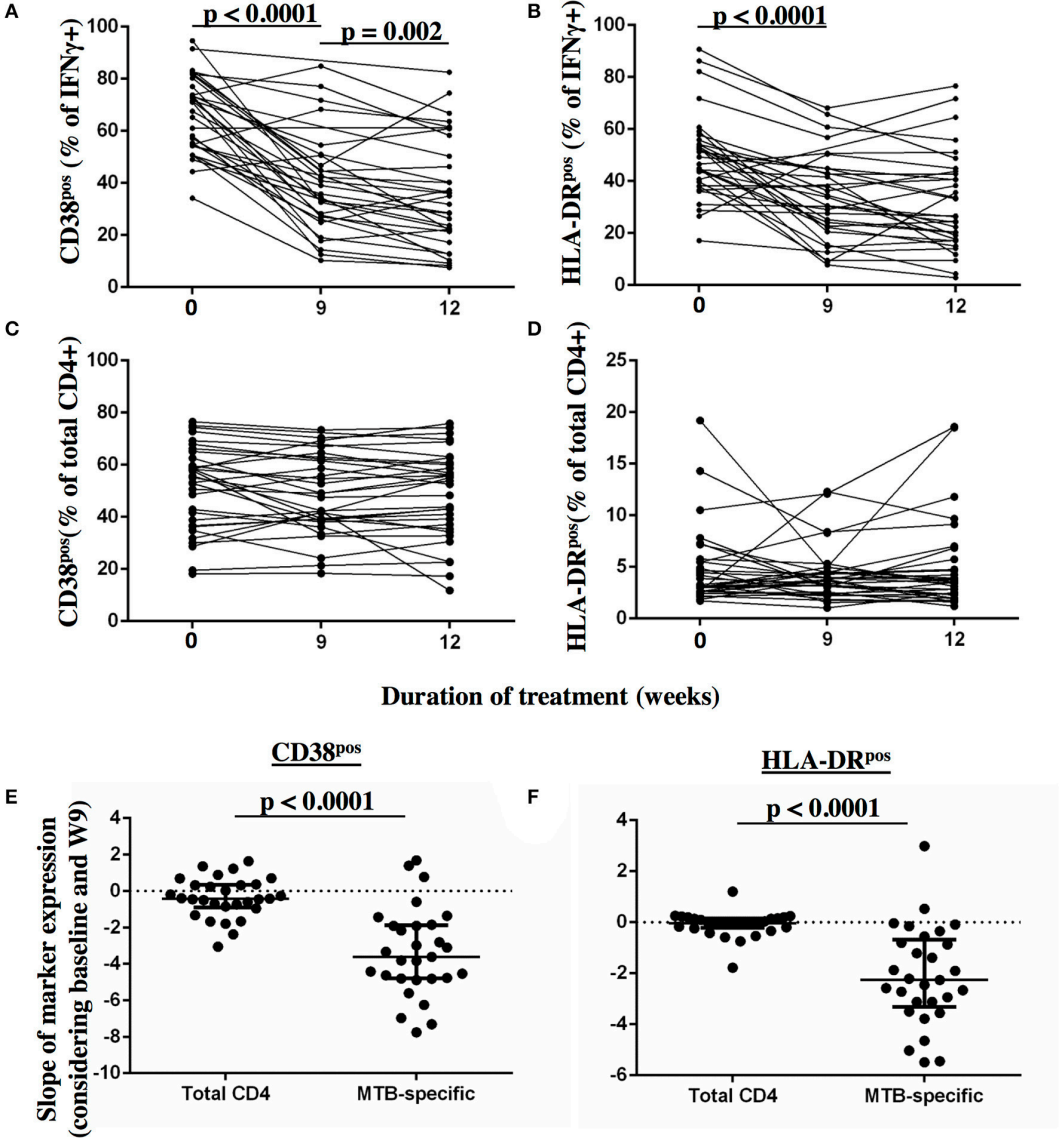
Detection of dynamic changes in CD38 and HLA-DR expression upon TB treatment initiation on MTB-specific, but not total CD4 T cells. The frequency of T cells expressing the activation markers CD38 and HLA-DR (y-axis) are shown for MTB-specific CD4 T cells **(A,B)** and for total CD4 T cells **(C,D)** before and at 9 and 12 weeks after treatment for each subject. MTB-specific CD4 T cells were characterized after PPD stimulation. The slopes of the activation marker expression on MTB-specific and on total CD4 T cells were compared for CD38 **(E)** and HLA-DR **(F)** between baseline and week 9 (*n* = 29). Statistical analyses for paired data were performed using the Wilcoxon-signed rank paired test. None-paired data analyzed using the Mann-Whitney test. *P*-values below 0.05 are indicated.

**Figure 5:**
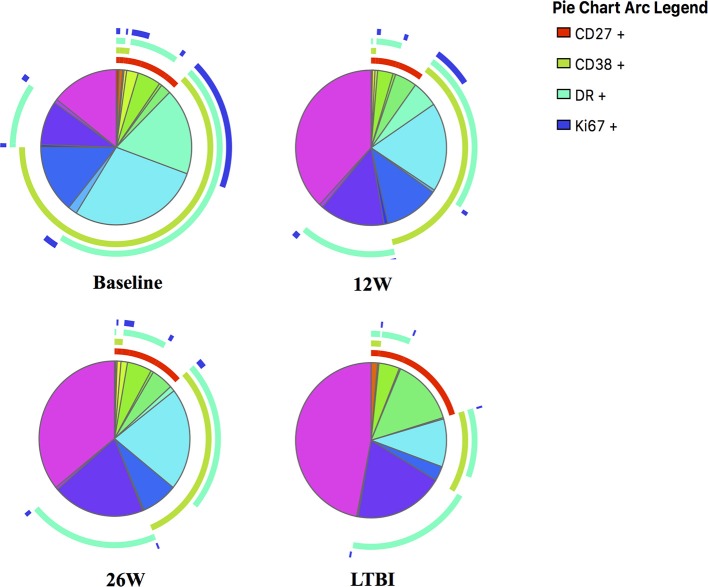
SPICE analyses for in-depth phenotypic profiling of MTB-specific CD4 T cells. Shown are SPICE pie charts visualizing the mean frequency for each of the 16 possible phenotypic profiles of MTB-specific CD4 T cells. The arcs indicate the proportion of cells that express CD27 (red), CD38 (green), HLA-DR (light blue) and/or Ki67 (dark blue). The time point or LTBI infection status is indicated below each pie chart.

Frequencies of activated MTB-specific CD4 T cells declined dramatically from pre-treatment to W9 post-treatment (*p* < 0.0001, Figure 3), while CD27^high^ cell frequencies remained largely unchanged (*p* = 0.8). These treatment-induced changes varied substantially between individuals and were exclusively observed in the MTB-specific T cell compartment, but not in the non-specific CD4 T cells (Figures 4A–D, Ki67 not shown). The numeric data for the underlying Figure 4 is provided in Supplementary Table 1. Consequently the changes in CD38 and HLA-DR expression until week 9 differed significantly between MTB-specific and total CD4 T cells (*p* < 0.0001 for both markers, Figures 4E,F). Only comparatively moderate changes were observed between W9 and W26 for CD38^pos^ and HLA-DR^pos^ cell frequencies. The median frequency of Ki67^pos^ cells declined further to 1.5% (*p* = 0.01) between W12 and 26, whereas the median frequency of CD27^low^ cells remained unchanged between before and at the end of treatment (median: 91%, Figures 3B,D). Using SPICE analyses (28) combinatorial changes of the 4 phenotypic markers on MTB-specific CD4 T cells were analyzed (Figure 5). The pie chart arcs show that TB treatment induced substantial reductions in MTB-specific T cell frequencies co-expressing the activation markers CD38, HLA-DR, and Ki67 in different combinations as shown.

### CD38 and CD27 expression phenotypes differentiate 3 MTB infection states: active TB disease, treated TB, and latent MTB infection

**Figure 6:**
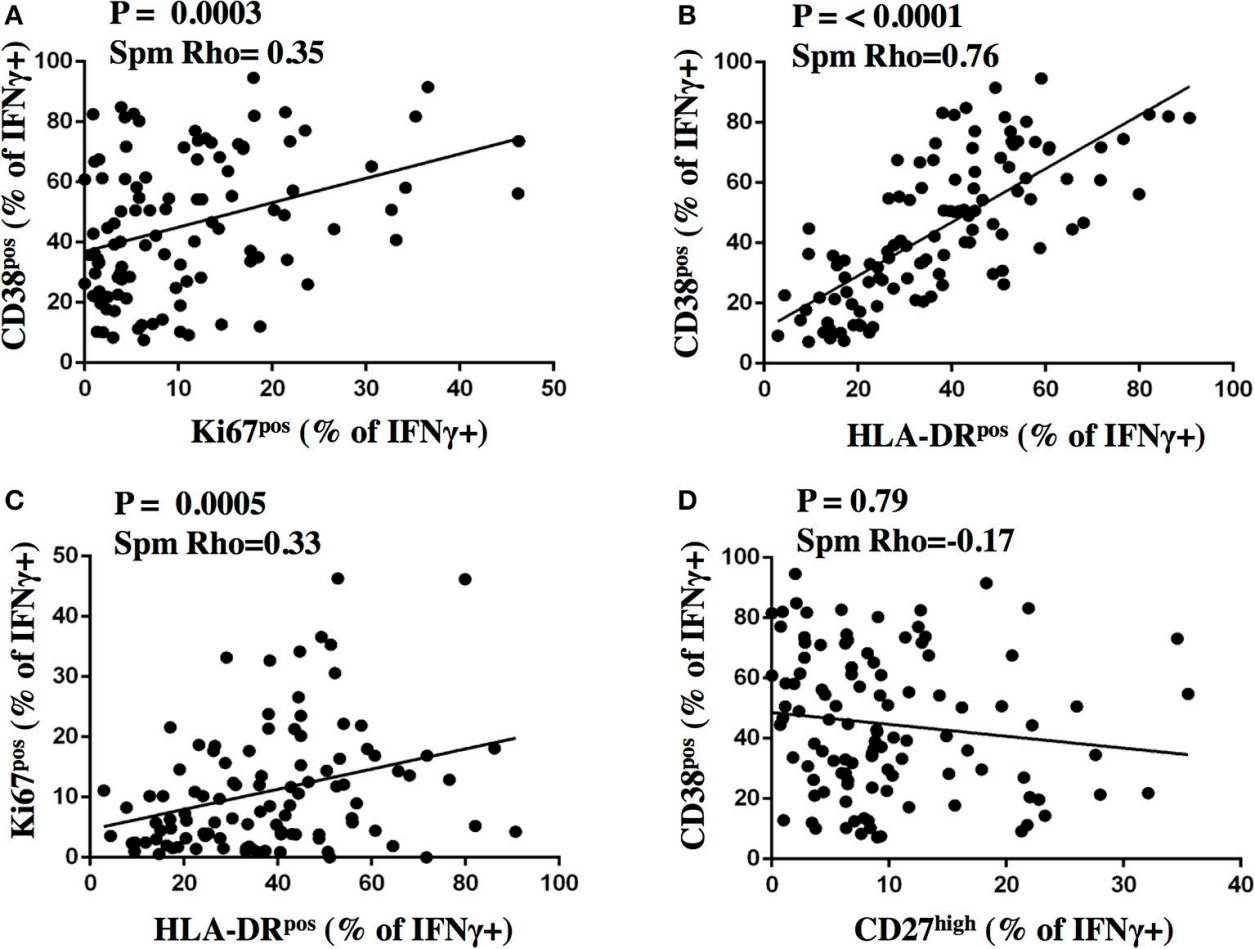
Correlation analysis of activation and maturation marker expression on MTB-specific CD4 T cells. The proportion of IFNγ+ MTB-specific CD4 T cells expressing activation and maturation markers after stimulation were plotted for CD38 and Ki67 **(A)**, CD38 and HLA-DR **(B)**, Ki67 and HLA-DR **(C)**, CD38 and CD27 **(D)** on the y- and x-axis, respectively for samples from subjects with aTB from before and after treatment initiation (*n* = 109). The Spearman's rank test was used for statistical analysis.

We next addressed the question whether and to what degree the expression of activation and maturation markers on MTB-specific CD4 T cells correlate with one another and analyzed data from aTB patients before and after TB treatment (*n* = 109 subject visits, Figure 6). The frequency of all the activation marker positive cells correlated with each other (all *p* < 0.0005). The strength of correlation varied; CD38 and HLA-DR expression correlated most strongly (Spm Rho = 0.76). The correlation of Ki67 with either CD38 or HLA-DR was comparatively weak (Spm Rho = 0.35 and 0.33, respectively). A weak inverse correlation was detected for the frequencies of CD27^high^ cells with HLA-DR^pos^ (data not shown, both *p* < 0.05). In contrast, frequencies of CD27^high^ MTB-specific CD4 T cells did not correlate with the frequencies of CD38^pos^ and Ki67^pos^ cells (Figure 6D and data not shown, *p* = 0.79 and *p* = 0.4, respectively). Hence, simultaneous assessment of CD38 and CD27 holds most information on TB disease and treatment status. Indeed, by assessing only these two markers, the phenotypic characteristics of MTB-specific CD4 T cells at week 26 still differed from LTBI with median frequencies of 28.8 and 12.1% for CD27^high^ (*p* = 0.0003) and CD38^pos^ cells (*p* = 0.008), respectively. Hence, phenotypic profiles differed significantly between aTB (profile: CD38^pos^, CD27^low^), treated TB (CD38^neg/pos^, CD27^low^), and LTBI (CD38^neg^, CD27^high^).

### Treatment induced reductions in TAM expression profiles on MTB-specific CD4T cells reflect declining bacterial burden in sputum

Using the cut-off of < 31.55% CD38^pos^ MTB-specific CD4 T cells to define LTBI, 37.5% (12 of 32) and 51.4% (18 of 35) of aTB patients had a TAM-TB profile consistent with “LTBI” at W9 and W12 post-treatment, respectively. To address whether such a phenotypic profile—determined at a single post-treatment time point—indicates *in vivo* mycobacterial clearance, we compared the time to last culture-positive result from these patients with this “cured TAM-TB assay profile” to those without such a TAM-TB profile. The two groups did not differ (*p* = 0.58, data not shown) and those with a “cured signature” still included 5 and 4 subjects, who were still culture positive at or after W9 and W12, respectively. Similar results were obtained using a more stringent cut off of < 19.7% CD38^pos^ MTB-specific CD4 T cells, which defined the lower quartile of CD38^pos^ cells at W12 (*p* = 0.26). Hence, a “cured” TAM-TB signature measured at a single post-treatment time point was not suggestive for clearance of viable bacteria in sputum nor did it differentiate rapid from slow treatment responders.

**Figure 7:**
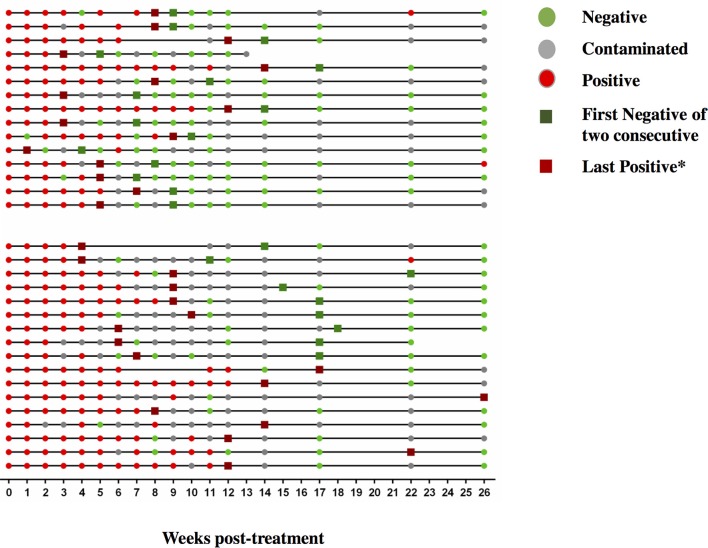
Sequential MGIT culture results. Sequential MGIT culture results from 17 study visits from week 0 to week 26 (x-axis) are shown for each subject with a TAM-TB assay result from baseline and/or at week 9 and/or week12 (*n* = 32). The upper and lower line graphs indicate culture results from subjects with ≤ 4 (*n* = 15) and ≥4 weeks (*n* = 17) between the last positive and stable culture conversion, respectively. Red dots indicate a MTB positive culture result, green dots a negative culture result, and gray dots indicate a contaminated sample. Dark green squares indicate stable culture conversion defined as the first of two consecutive culture negative results. Dark red square*: last culture positive sample before 2 consecutive culture negative samples.

**Figure 8:**
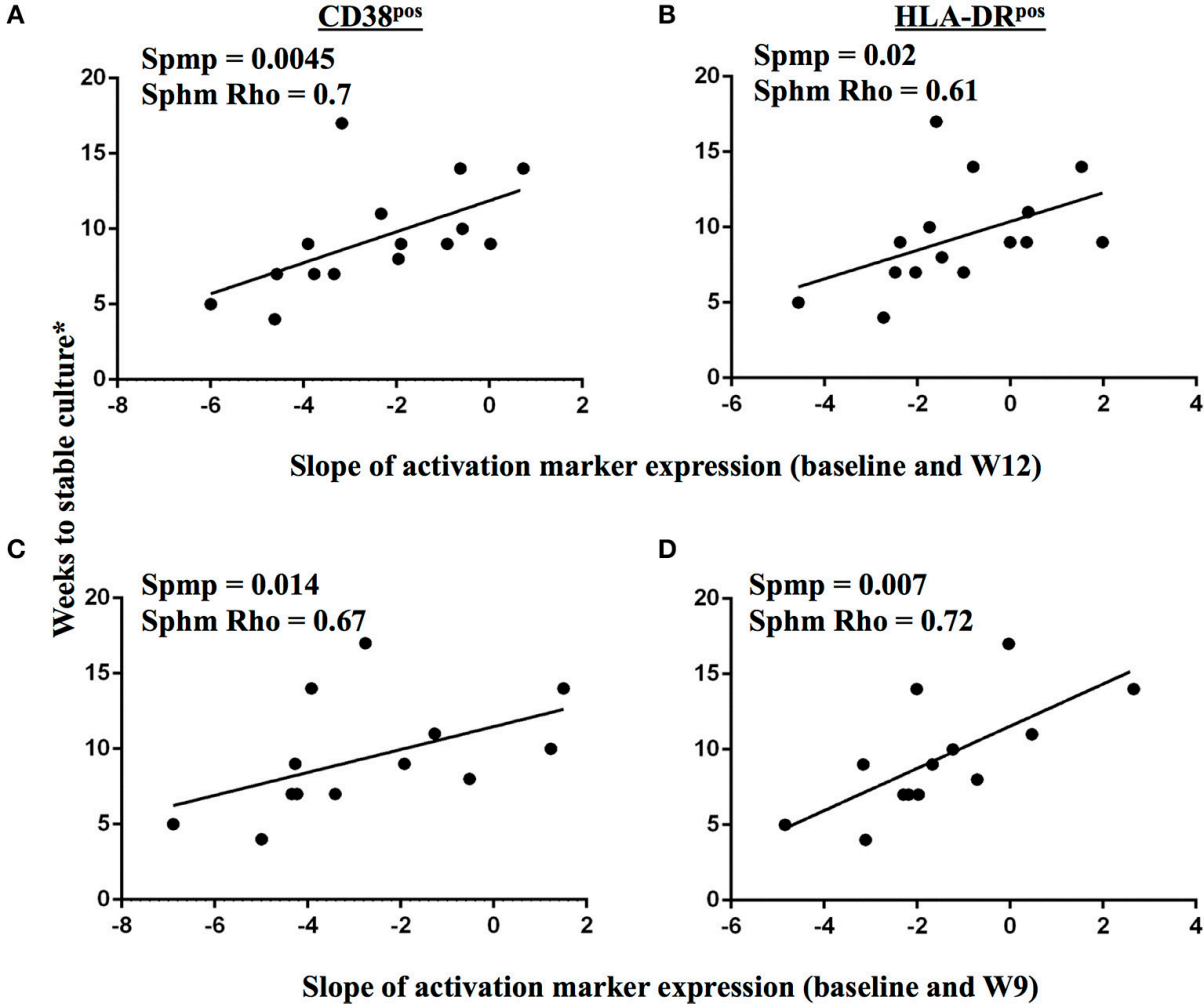
Changes in TAM expression profiles on MTB-specific CD4 T cells upon treatment initiation reflect declining bacterial burden in sputum. A correlation analysis between time to stable culture conversion and the slope of CD38 and HLA-DR marker expression dynamics on IFNγ+ MTB-specific CD4 T cells is shown for the time interval from baseline to week 12 **(A,B**, *n* = 15), and from baseline to week 9 **(C,D**, *n* = 13), respectively, for subjects with accurately defined time point of less than 5 weeks between the last positive MGIT culture result and stable culture conversion. The Spearman's rank test was used for statistical analysis.

We next addressed whether changes in the frequencies of T cell activation marker positive MTB-specific CD4 T cells between baseline and W9; and between baseline and W12 were linked to treatment-induced bacterial clearance using the primary PanACEA study endpoint—time to stable culture negativity. 32 of 39 subjects had TAM-TB results at baseline and at W12 and/or W9 and thus the slope for the change in expression of the individual TAMs after treatment initiation could be determined. 15 of these 32 subjects also had an accurate endpoint determination of ≤ 4 weeks between the last positive and stable culture conversion (Figure 7). In the other 17 subjects, determination of the accurate time to culture conversion was compromised due to increasing rates of culture contamination as treatment progressed—a common phenomenon (29). These either did not reach stable culture conversion or had large gaps between the last positive and stable culture conversion and hence were excluded from analyses. Taking into consideration baseline and W12 results, the slope of decline in expression of CD38 (*p* = 0.0045, Rho = 0.7) and HLA-DR (*p* = 0.02, Rho = 0.61) on MTB-specific CD4 T cells correlated with the time to stable culture conversion (Figures 8A,B). Likewise, similar correlations were detected when considering measurements at baseline and week 9 (*n* = 13 subjects, CD38: *p* = 0.015, Rho = 0.67; HLA-DR: *p* = 0.007, Rho = 0.72, Figures 8C,D). No such correlations were detected for CD38 and HLA-DR expression on total CD4 T cells (data not shown).

## Discussion

Substantial reductions in the expression of activation markers on MTB-specific CD4 T cells were observed at W9 into TB treatment for most patients. In contrast, expression of these markers on total CD4 T cells remained comparable to pre-treatment values, showing that TB treatment-induced changes in T cell activation status are specific to the MTB-specific T cell compartment. Individual patients varied substantially in the early reduction in MTB-specific CD4 T cell activation until week 9 and week 12; and for both of these time intervals, the degree of change in activation correlated well with the mycobacterial response. Our data therefore provide evidence that early changes in TAM expression profiles on MTB-specific CD4 T cells reflect clearance of viable mycobacteria *in vivo*. These TAM-TB profiles may therefore serve as a surrogate marker for treatment efficacy, as originally proposed by Adekambi et al. (5) and now further substantiated in more aTB patients with more precisely determined time to culture conversion. This immunological approach therefore bypasses the issues associated with the collection of sputum specimen (e g., in young children, after TB treatment initiation) and increased culture contamination rates as treatment progresses (29); which renders treatment monitoring by serial cultures difficult and often imprecise on a per person level, as was observed in our patient subset. It is noteworthy, that all tested TB patients responded to the MTB antigen PPD, therefore enabling the determination of the activation status on MTB-specific CD4 T cells.

Shortening treatment is a major objective of TB drug development and has been tested in 4 recent studies (20–23); the majority (at least 80%) of patients treated in these trials for only 4 months had been cured without relapse within the defined follow up of 18 months; but nevertheless the trials were declared unsuccessful due to high relapse rates in the 4 months regimens. While speculative at this point, a TAM-TB assay approach based treatment-monitoring algorithm could therefore potentially help to personalize TB treatment duration by discrimination of patients who respond well to TB treatment marked by a substantial reduction in MTB-specific CD4 T cell activation—and those who do not.

The dynamic changes in expression of T cell activation markers observed upon treatment initiation contrasted with the minor changes observed for the maturation marker CD27. We assessed, which combination of markers are most informative on treatment and disease/infection status. Consistent with previous results (5), expression of CD38 differentiated best between aTB and LTBI. The three activation markers correlated with one another and thus the determination of one activation marker—CD38—might suffice to differentiate active TB from LTBI and to monitor the treatment response. No correlation was detected between expression of CD38 and CD27 and the latter best differentiated treated TB at W26 from LTBI. Hence, simultaneous assessment of CD38 and CD27 expression on MTB-specific CD4 T cells, can help to differentiate between three MTB infection/disease states; (1) aTB—defined by high expression of activation markers, but low CD27 expression; (2) LTBI—defined by low expression of activation markers and high expression of CD27 and (3) treated TB—defined by low expression of activation markers and continuously low CD27 expression. Our data therefore support the concept that phenotypic characteristics of pathogen-specific T cells may not only differentiate between active TB and LTBI, but may also detect past episodes of pathogen activity, which were resolved through treatment or naturally.

Previous reports on CD27 expression on MTB-specific CD4 T cells also showed no substantial increase by 26 weeks of treatment (6). However, at 12 months after the end of TB treatment it appears to revert to levels characteristic for LTBI (8). It could be that residual TB disease activity at the end of TB treatment and during LTBI, might contribute to such a CD27^low^ profile, as suggested by a recent positron emission tomography (PET-CT) study. This study showed persistent, active lesion activity in the majority of cured TB patients even until 6 months after TB treatment (30). A large range of bacterial loads and metabolic activity in MTB lesions (31–33) has also been observed in non-human primates with LTBI consistent with a broad spectrum between LTBI, treated and active disease (34, 35). We had previously reported on a HIV seroconverter where CD27 downregulation on MTB-specific T cells preceded active TB diagnosis by 6–9 months (2). It would therefore be of great interest to better define the range of expression of phenotypic markers on MTB-specific T cells and other host response markers (36–38) in relation to the presence and dynamics of TB lesions *in vivo* in subjects with LTBI and in patients after treatment initiation (30) with systematic follow up on active TB disease progression, and treatment failure and relapse, respectively.

It is known that males account to 65% of aTB patients globally (39). Our LTBI control group consisted only of females, which raised the concern whether gender might have confounded our results. To address this question, males (or females) were excluded in a sub-group analysis (Supplementary Figure 2); there was no overlap of CD38 and Ki67 in subjects with LTBI and aTB, when considering only females (*p* < 0.0001). Further, no gender-associated difference in the TAM-TB profile at baseline was detected, whereas significant reductions in T cell activation marker expression—but not for CD27 expression—were induced by TB treatment regardless of gender. Hence there was no evidence that gender alone influenced the TAM-TB profile of MTB-specific CD4 T cells in our study. It is however noteworthy that males had significantly higher frequencies of proliferating (Ki67+) MTB-specific CD4 T cells and a trend for increased expression of CD38 at 12 weeks post treatment initiation (*p* = 0.09) compared to females (data not shown). The latter finding is “consistent” with another finding of the Panacea-TB MAMS treatment study; females cleared mycobacteria significantly faster upon treatment initiation as compared to males (unpublished results). Hence, gender-associated differences in activation marker profiles of MTB-specific CD4 T cells at 12 weeks after treatment initiation probably reflect gender-related differences in the rate of mycobacterial clearance.

Limitations in this study were the lack of available X-ray scores as well as disease severity assessment. We were also not able to study the effect of HIV infection on TAM-TB assay profiles during treatment monitoring, because our cohort recruited exclusively HIV negative patients. HIV infection is associated with increased levels of systemic T cell activation (40, 41). Another study investigated the effect of HIV on MTB-specific CD4 T cell activation, and maturation, showing only little influence (7). Larger studies in well-characterized cohorts need to clarify the influence of these parameters on TAM-TB assay results and their changes upon treatment initiation. While the need for flow cytometry and basic cell culture methods may limit the use of this approach in resource poor settings, our data show that more complex flow cytometry is in principle not needed. We and others have previously shown that by using whole blood this assay can be simplified substantially (2, 42). Other methods assessing the MTB-specific cytokine secretion upon whole blood stimulations may also allow specific diagnoses of active TB (43), whereas monitoring the TB treatment response using the latter approach so far appears less promising (44). The measurement of soluble biomarkers in the blood without prior stimulation also may hold valuable information on TB disease and treatment status (45). The diagnostic value of these needs further investigation.

In conclusion, our data on using phenotypic profiles of MTB-specific CD4 T cells as a surrogate marker for treatment efficiency warrant further research and methodological simplification to define its usefulness in standard clinical sttings and during TB drug trials.

## Author contributions

All authors contributed to manuscript writing. NN, GK, BM, HS, SM, and CM were involved in design, conduct, and analysis of the underlying patient study at both participating clinical study centers. A-MM, MB, SG, MH, and NH conceived, planned, and managed the patient study from the study sponsor side. MA, MH, NH, ES, UB, IK, RL, and CG participated in data analysis. MA, KH, MC, AH, and CG contributed to experimental work. MH, NH, and CG conceived the immunological sub-study reported here.

### Conflict of interest statement

The authors declare that the research was conducted in the absence of any commercial or financial relationships that could be construed as a potential conflict of interest. The reviewer RT and handling Editor declared their shared affiliation.
