# Supplementary material for: Phenotypic Changes on Mycobacterium Tuberculosis-Specific CD4 T Cells as Surrogate Markers for Tuberculosis Treatment Efficacy
Source: Front Immunol. 2018 Sep 28;9:2247. doi: 10.3389/fimmu.2018.02247 (PMC6172348; doi:10.3389/fimmu.2018.02247)
Supplement: Supplementary file 2 [file Data_Sheet_1.PDF]

Supplementary Figures

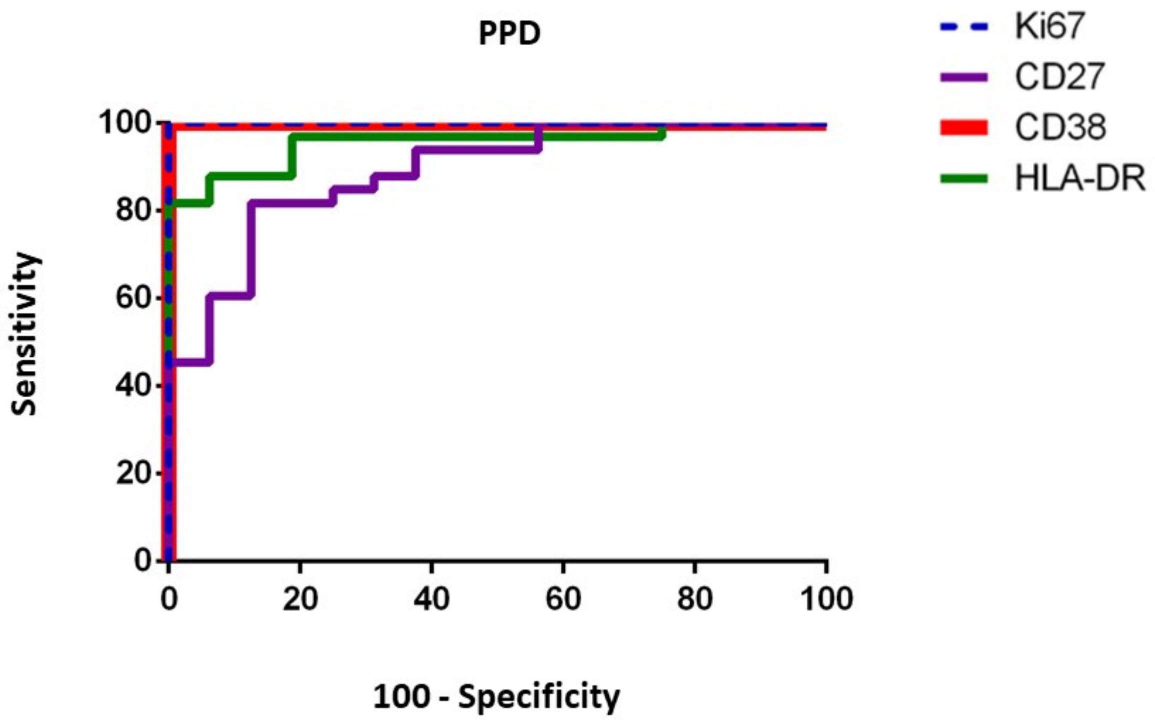

Supplementary Figure 1: Receiver Operating Characteristic (ROC) analysis to differentiate aTB and LTBI by using individual activation and maturation markers. ROC curves comparing the frequency of IFNγ+ MTB-specific CD4 T cells expressing activation markers Ki67 (blue dotted line), CD38 (red line), HLA-DR (green line) and those expressing the maturation marker CD27 (purple line) in subjects with aTB before treatment initiation (n= 33) and endemic controls with LTBI (n= 16). MTB-specific CD4 T cells were characterized after PPD stimulation.

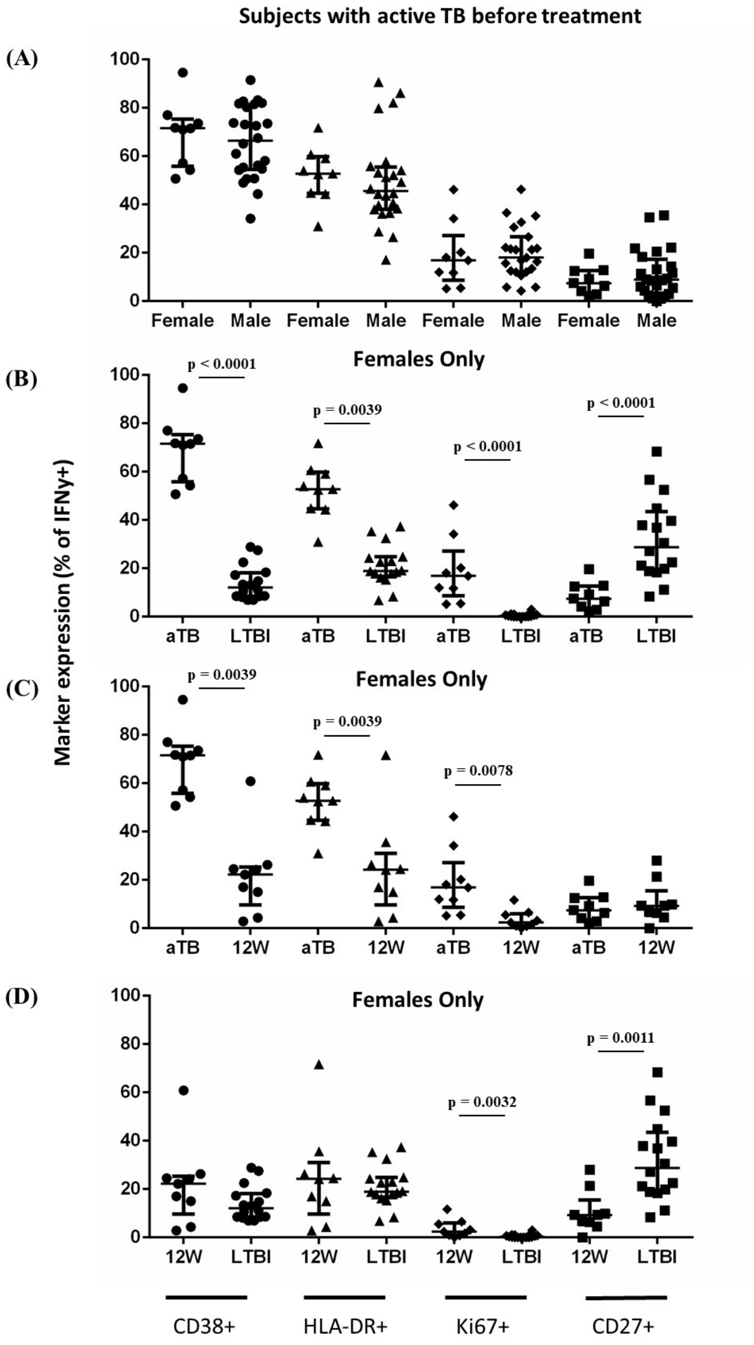

Supplementary Figure 2. Phenotypic profiles of MTB-specific CD4 T cells in males and females with aTB, after TB treatment initiation and during LTBI. The frequency of MTB-specific CD4 T cells expressing the activation markers CD38, HLA-DR, Ki67 and the maturation marker CD27 is shown on the y-axis for (A) pulmonary TB patients as stratified by sex and (B) for females only stratified by MTB infection status, (C) before and 12 weeks after TB treatment initiation and (D) a comparison between the TAM profile at 12W into treatment and LTBI. MTB-specific CD4 T cells were characterized after PPD stimulation. Statistical analyses were performed using the Mann-Whitney test. Median values, interquartile range and p-values below 0.05 are indicated.
